# Supplementary material for: Capturing Compensatory Reserve in Sarcopenia: A Bioengineering Framework for Multidimensional Temporal Analysis of Center-of-Pressure Signals
Source: Bioengineering (Basel). 2025 Oct 23;12(11):1143. doi: 10.3390/bioengineering12111143 (PMC12649375; doi:10.3390/bioengineering12111143)
Supplement: Supplementary file 1 [file bioengineering-12-01143-s001.zip › A.1.Detailed calculation of kinematic formulas .pdf]

S1. Kinematic Features Classification, Included Metrics and Formulas

Category	Features	Formulas
Displacement Parameters	X$_n$<br>Y$_n$<br>R$_n$<br>COV<br>MEAN$_X$<br>MEAN$_Y$<br>MEAN_DIST$_X$<br>MEAN_DIST$_Y$<br>MEAN_DIST	$X_n = \text{COP-CX}_n - \overline{\text{COP-CX}}$<br>$Y_n = \text{COP}-\text{CY}_n - \overline{\text{COP}-\text{CY}}$<br>$R_n = \sqrt{X_n^2 + Y_n^2}$<br>$COV = \frac{\sum (X_n \cdot Y_n)}{N}$<br>$MEAN_X = \overline{X_n}$<br>$MEAN_Y = \overline{Y_n}$<br>$MEAN\_DIST_X = \overline{\lvert X_n \rvert}$<br>$MEAN\_DIST_Y = \overline{\lvert Y_n \rvert}$<br>$MEAN\_DIST = \overline{R_n}$
Sway Amplitude Metrics	MAX$_X$<br>MAX$_Y$<br>MAX RADIUS<br>RMS$_X$<br>RMS$_Y$<br>RMS RADIUS<br>RANGE$_X$<br>RANGE$_Y$<br>RANGE_XY<br>RANGE<br>RATIO<br>PLANAR$_{DEV}$<br>SWAY LENGTH$_X$<br>SWAY LENGTH$_Y$<br>SWAY_ LENGTH<br>AREA PER SEC<br>COEF SWAY$_{DIR}$	$MAX_X = \max(\lvert X_n \rvert)$<br>$MAX_Y = \max(\lvert Y_n \rvert)$<br>$MAX\_RADIUS = \max(R_n)$<br>$RMS_X = \sqrt{\overline{X_n^2}}$<br>$RMS_Y = \sqrt{\overline{Y_n^2}}$<br>$RMS\_RADIUS = \sqrt{\overline{R_n^2}}$<br>$RANGE_X = \max(X_n) - \min(X_n)$<br>$RANGE_Y = \max(Y_n) - \min(Y_n)$<br>$RANGE\_XY = \sqrt{RANGE_X^2 + RANGE_Y^2}$<br>$RANGE\_RATIO = \frac{RANGE_X}{RANGE_Y}$

		$PLANAR_{DEV} = \sqrt{RMS_X^2 + RMS_Y^2}$<br>$SWAY\_LENGTH_X = \sum \lvert \Delta X_n \rvert$<br>$SWAY\_LENGTH_Y = \sum \lvert \Delta Y_n \rvert$<br>$SWAY\_LENGTH = \sqrt{\sum(\Delta X_n^2) + \sum(\Delta Y_n^2)}$<br>$AREA\ PER\ SEC. = \frac{1}{2T}\sum_{n=1}^{N-1}\lvert X_{n+1}Y_n - X_nY_{n+}$ [illegible]<br>$COEF\_SWAY_{DIR} = \frac{COV}{RMS_X \cdot RMS_Y}$
Dynamic Characteristics	VELOCITYXL, VELOCITYXR, VELOCITYYL, VELOCITYYR, VELOCITYX, VELOCITYY, VELOCITY, ACCXL, ACCYR, ACCYL, ACCYR, ACCX, ACCY, ACC, MEANVELX, MEANVELY, MEANVEL, STDVELX, STDVELY, STDVEL	$VELOCITY_{X_L} = \left\lvert \frac{\Delta COP - LX_n}{\Delta t} \right\rvert$<br>$VELOCITY_{X_R} = \left\lvert \frac{\Delta COP - RX_n}{\Delta t} \right\rvert$<br>$VELOCITY_{Y_L} = \left\lvert \frac{\Delta COP - LY_n}{\Delta t} \right\rvert$<br>$VELOCITY_{Y_R} = \left\lvert \frac{\Delta COP - RY_n}{\Delta t} \right\rvert$<br>$VELOCITY_X = \left\lvert \frac{\Delta COP - CX_n}{\Delta t} \right\rvert$<br>$VELOCITY_Y = \left\lvert \frac{\Delta COP - CY_n}{\Delta t} \right\rvert$<br>$VELOCITY = \sqrt{VELOCITY_X^2 + VELOCITY_Y^2}$<br>$ACC_{X_L} = \left\lvert \frac{\Delta VELOCITY_{X_L}}{\Delta t} \right\rvert$<br>$ACC_{X_R} = \left\lvert \frac{\Delta VELOCITY_{X_R}}{\Delta t} \right\rvert$<br>$ACC_{Y_L} = \left\lvert \frac{\Delta VELOCITY_{Y_L}}{\Delta t} \right\rvert$<br>$ACC_{Y_R} = \left\lvert \frac{\Delta VELOCITY_{Y_R}}{\Delta t} \right\rvert$<br>$ACC_X = \left\lvert \frac{\Delta VELOCITY_X}{\Delta t} \right\rvert$

		$ACC_Y = \left\| \frac{\Delta VELOCITY_Y}{\Delta t} \right\|$<br>$ACC = \sqrt{ACC_X^2 + ACC_Y^2}$<br>$MEAN_{VEL_X} = \frac{SWAY\_LENGTH_X}{T}$<br>$MEAN_{VEL_Y} = \frac{SWAY\_LENGTH_Y}{T}$<br>$MEAN_{VEL} = \frac{SWAY\_LENGTH}{T}$<br>$STD_{VEL_X} = \sqrt{(VELOCITY_X - \overline{VELOCITY_X})^2}$<br>$STD_{VEL_Y} = \sqrt{(VELOCITY_Y - \overline{VELOCITY_Y})^2}$<br>$STD_{VEL} = \sqrt{(VELOCITY - \overline{VELOCITY})^2}$
Symmetry Indices	SYMMETRY_ RANGE$_{XY}$	$\mathrm{SYMMETRY\_RANGE}_{XY} = \frac{1}{2}(\left\|\mathrm{COP-LX}_n - \mathrm{COP-RX}_n\right\| + \left\|\mathrm{COP-LY}_n -$

All these formulas can be edited in MathType within Word.
