# Supplementary material for: Capturing Compensatory Reserve in Sarcopenia: A Bioengineering Framework for Multidimensional Temporal Analysis of Center-of-Pressure Signals
Source: Bioengineering (Basel). 2025 Oct 23;12(11):1143. doi: 10.3390/bioengineering12111143 (PMC12649375; doi:10.3390/bioengineering12111143)
Supplement: Supplementary file 1 [file bioengineering-12-01143-s001.zip › A.2. Hyperparameter search ranges for machine learning algorithms .pdf]

S2. Hyperparameter search ranges for machine learning algorithms

Model	Parameters	Optimal parameter
KNN	n_neighbors: [3, 5, 7, 13]<br>weights: ['distance']	n_neighbors=13,<br>weights='distance'
SVM	C: [0.1, 0.5, 1]<br>kernel: ['rbf']<br>gamma: ['scale']	C=0.5,<br>kernel='rbf',<br>gamma='scale'
Random Forest	n_estimators: [50, 80, 100]<br>max_depth: [3, 5, None]	n_estimators=80,<br>max_depth=5
Extra Trees	n_estimators: [50, 80, 100]<br>max_depth: [3, 5, None]	n_estimators=80,<br>max_depth=5
Logistic Regression	C: [0.1, 0.3, 0.5, 1]<br>penalty: ['l2']<br>max_iter: [1000]	C=0.3,<br>penalty='l2',<br>max_iter=1000
Decision Tree	max_depth: [3, 4, 5, None]<br>min_samples_split: [10]	max_depth=4, min_sample<br>s_split=10
Naive Bayes	GaussianNB (var_smoothing=1e-9<br>priors=None)	GaussianNB<br>(var_smoothing=1e-9, 1e-<br>8, 1e-7,<br>priors=None)
