# Supplementary material for: Capturing Compensatory Reserve in Sarcopenia: A Bioengineering Framework for Multidimensional Temporal Analysis of Center-of-Pressure Signals
Source: Bioengineering (Basel). 2025 Oct 23;12(11):1143. doi: 10.3390/bioengineering12111143 (PMC12649375; doi:10.3390/bioengineering12111143)
Supplement: Supplementary file 1 [file bioengineering-12-01143-s001.zip › A.6. SHAP visualizations for kinematic features.pdf]

S6. SHAP visualizations for kinematic features

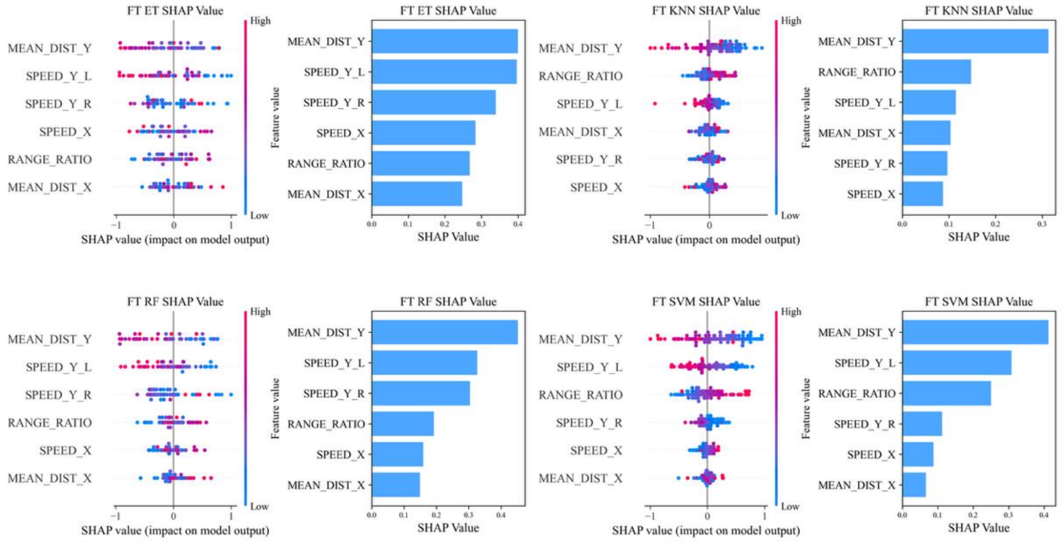

Figure S6-1： SHAP Feature Importance Distribution of Kinematic Features in Feet-Together (FT) Stance

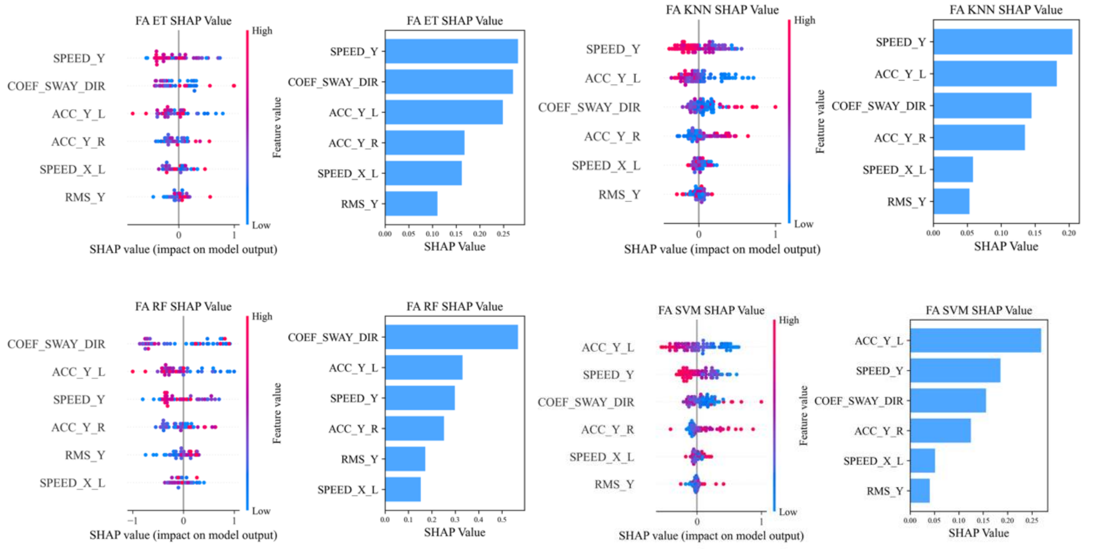

Figure S6-2： SHAP Feature Importance Distribution of Kinematic Features in Feet-Apart (FA) Stance

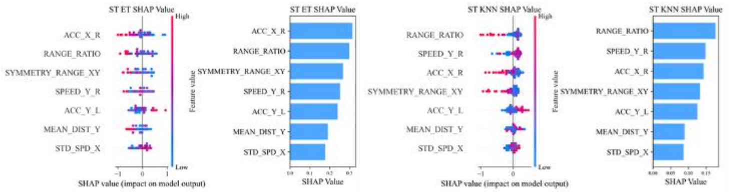

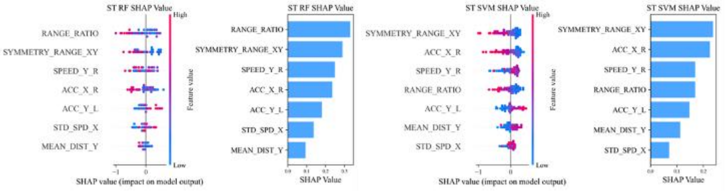

Figure S6-3：SHAP Feature Importance Distribution of Kinematic Features in Semi-tandem (ST) Stance
