# Supplementary material for: Capturing Compensatory Reserve in Sarcopenia: A Bioengineering Framework for Multidimensional Temporal Analysis of Center-of-Pressure Signals
Source: Bioengineering (Basel). 2025 Oct 23;12(11):1143. doi: 10.3390/bioengineering12111143 (PMC12649375; doi:10.3390/bioengineering12111143)
Supplement: Supplementary file 1 [file bioengineering-12-01143-s001.zip › A.7. Additional SHAP visualizations for multidimensional temporal features.pdf]

S7. Additional SHAP visualizations for multidimensional temporal features

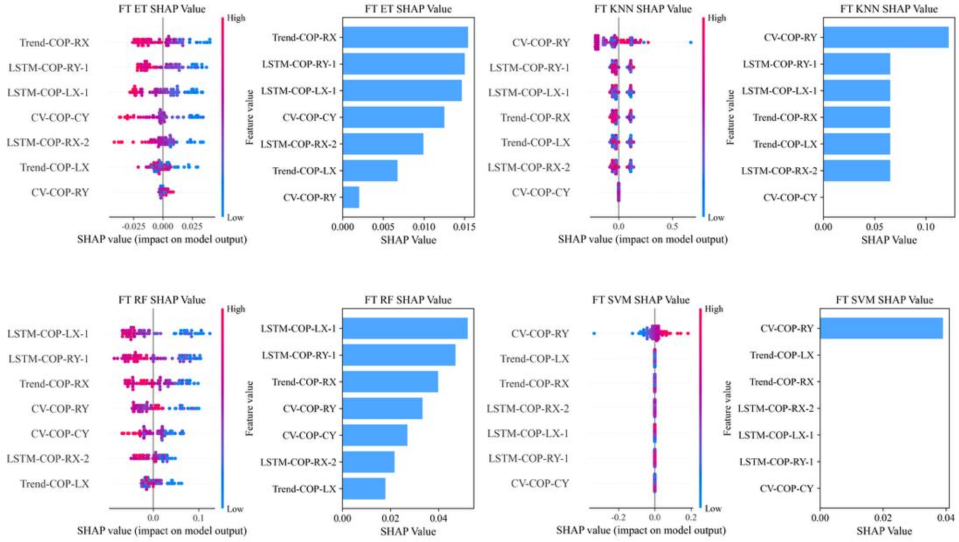

Figure S7-1： SHAP Summary Plot of Multidimensional Temporal Features in Feet-Together (FT) Stance

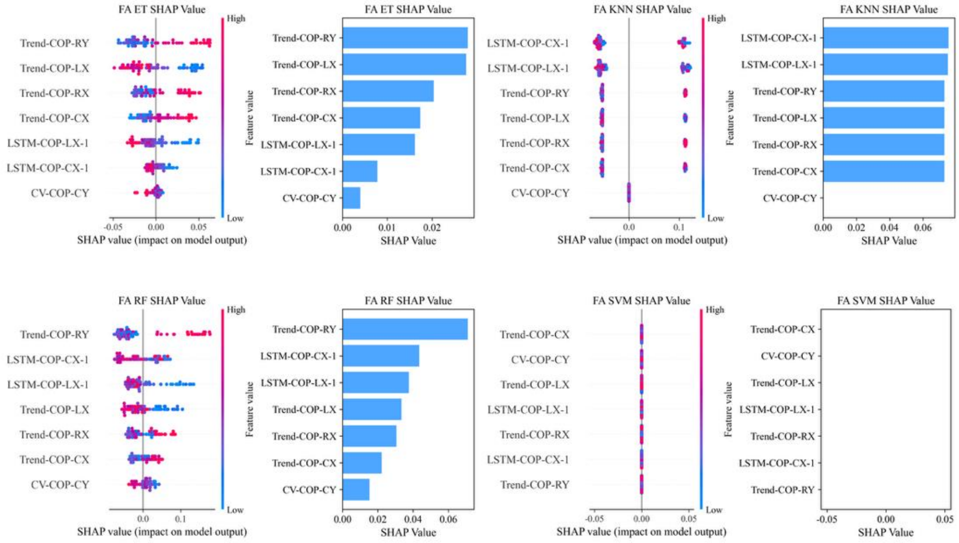

Figure S7-2： SHAP Summary Plot of Multidimensional Temporal Features in Feet-Apart (FA) Stance
