# Supplementary material for: Capturing Compensatory Reserve in Sarcopenia: A Bioengineering Framework for Multidimensional Temporal Analysis of Center-of-Pressure Signals
Source: Bioengineering (Basel). 2025 Oct 23;12(11):1143. doi: 10.3390/bioengineering12111143 (PMC12649375; doi:10.3390/bioengineering12111143)
Supplement: Supplementary file 1 [file bioengineering-12-01143-s001.zip › A.8. Parameter optimization for DTW (γ selection) and LSTM architecture benchmarking.pdf]

## S8. Parameter optimization for DTW (γ selection) and LSTM architecture benchmarking

Grid search optimized DTW's γ (Table S8-1), and LSTM unit size was benchmarked (Table S8-2):

**Table S8-1. DTW parameter (γ) optimization results**

| γ | F1-score (mean±SD) | ROC-AUC (mean±SD) |
|---|---|---|
| 0.5 | 0.74±0.06 | 0.73±0.09 |
| 1 | 0.75±0.07 | 0.74±0.08 |
| 1.5 | 0.78±0.06 | 0.76±0.09 |
| 2 | 0.72±0.09 | 0.71±0.10 |

γ=1.5 outperformed other values (F1: $p$<0.01 vs γ=1.0, $p$<0.001 vs γ=2.0; ROC-AUC: $p$=0.01 vs γ=2.0) with comparable efficiency, so it was selected.

**Table S8-2. Performance and computational efficiency of LSTM with varying unit sizes**

| LSTM units | F1-score (mean±SD) | ROC-AUC (mean±SD) | Total runtime (s) |
|---|---|---|---|
| 2 | 0.78±0.06 | 0.76±0.09 | 500.75 |
| 4 | 0.79±0.05 | 0.77±0.08 | 680.95 |
| 8 | 0.80±0.03 | 0.80±0.05 | 951.40 |
| 16 | 0.82±0.04 | 0.83±0.07 | 1802.70 |
| 32 | 0.81±0.08 | 0.82±0.07 | 2904.30 |

The 16-unit model achieved the highest performance, but the 2-unit model showed comparable metrics ($p$>0.05 vs 16-unit) with 3.6× faster runtime ($p$<0.001). The 32-unit model was inefficient and not superior to 16-unit, so 2-unit was chosen.

Optimized parameters (γ=1.5, 2-unit LSTM) were used for subsequent analyses.
