# Supplementary material for: Capturing Compensatory Reserve in Sarcopenia: A Bioengineering Framework for Multidimensional Temporal Analysis of Center-of-Pressure Signals
Source: Bioengineering (Basel). 2025 Oct 23;12(11):1143. doi: 10.3390/bioengineering12111143 (PMC12649375; doi:10.3390/bioengineering12111143)
Supplement: Supplementary file 1 [file bioengineering-12-01143-s001.zip › A.9. Complete classification performance of _all-deviation_ framework.pdf]

S9. Complete classification performance of "all-deviation" framework

Posture	Model	Accuracy	Accuracy_std	Precision	Precision_std	Recall	Recall_std	F1 score	F1 score _std	ROC-AUC	ROC-AUC -std	AUPR	AUPR _std
FT	KNN	1.5	0.80	0.06	0.82	0.09	0.80	0.22	0.78	0.10	0.79	0.07	0.77
FT	ET	1.5	0.74	0.04	0.77	0.12	0.75	0.19	0.73	0.08	0.70	0.06	0.69
FT	RF	1.5	0.74	0.04	0.69	0.06	0.89	0.11	0.77	0.04	0.72	0.04	0.73
FT	LR	1.5	0.67	0.05	0.64	0.07	0.87	0.17	0.72	0.05	0.63	0.07	0.63
FT	SVM	1.5	0.73	0.04	0.87	0.14	0.62	0.21	0.68	0.08	0.74	0.06	0.77
FT	NB	1.5	0.67	0.05	0.69	0.10	0.71	0.19	0.67	0.08	0.64	0.10	0.62
FT	DT	1.5	0.62	0.05	0.59	0.04	0.80	0.13	0.67	0.06	0.57	0.12	0.57
FA	KNN	1.5	0.78	0.12	0.87	0.11	0.69	0.30	0.72	0.23	0.75	0.20	0.80
FA	ET	1.5	0.75	0.06	0.92	0.11	0.55	0.10	0.68	0.08	0.72	0.12	0.78
FA	RF	1.5	0.72	0.05	0.73	0.10	0.76	0.21	0.72	0.08	0.71	0.11	0.75
FA	LR	1.5	0.66	0.06	0.70	0.16	0.71	0.17	0.68	0.04	0.62	0.07	0.66
FA	SVM	1.5	0.73	0.07	0.79	0.15	0.71	0.12	0.72	0.02	0.69	0.12	0.75
FA	NB	1.5	0.70	0.07	0.85	0.16	0.60	0.22	0.66	0.09	0.62	0.10	0.70
FA	DT	1.5	0.65	0.11	0.66	0.14	0.78	0.20	0.69	0.08	0.57	0.14	0.64
ST	KNN	1.5	0.83	0.03	0.81	0.05	0.85	0.04	0.83	0.03	0.83	0.05	0.82
ST	ET	1.5	0.78	0.07	0.75	0.07	0.85	0.09	0.80	0.06	0.81	0.06	0.82
ST	RF	1.5	0.81	0.04	0.83	0.09	0.80	0.11	0.81	0.05	0.82	0.04	0.84
ST	LR	1.5	0.74	0.03	0.70	0.05	0.85	0.07	0.76	0.01	0.75	0.04	0.76
ST	SVM	1.5	0.78	0.03	0.79	0.07	0.78	0.07	0.78	0.02	0.78	0.03	0.77
ST	NB	1.5	0.73	0.04	0.70	0.06	0.82	0.06	0.75	0.03	0.74	0.06	0.74
ST	DT	1.5	0.76	0.03	0.77	0.03	0.75	0.09	0.76	0.04	0.74	0.03	0.70
